# Polysulfated Trehalose as a Novel Anticoagulant Agent with Dual Mode of Action

**DOI:** 10.1155/2015/630482

**Published:** 2015-03-17

**Authors:** Qudsia Rashid, Mohammad Abid, Neha Gupta, Tarun Tyagi, Mohammad Z. Ashraf, Mohamad Aman Jairajpuri

**Affiliations:** ^1^Protein Conformation and Enzymology Lab, Department of Bio-Sciences, Jamia Millia Islamia (A Central University), Jamia Nagar, New Delhi 110 025, India; ^2^Medicinal Chemistry Lab, Department of Biosciences, Jamia Millia Islamia, New Delhi 110 025, India; ^3^Defense Institute of Physiology & Allied Sciences, Timarpur, Delhi 110 054, India

## Abstract

Physiological hemostatic balance is a coordinated outcome of counteracting coagulation and fibrinolytic systems. An imbalance of procoagulant and anticoagulant factors may result in life threatening thromboembolism. Presently, anticoagulant administration is the first line of therapy for the treatment of these conditions and several anticoagulants have been approved, including various forms of heparin. However, the polyanionic nature and multispecificity of heparin pose several complications. Generally, the polysulfated compounds with antithrombotic potential are thought to have feasible synthetic procedures with much less bleeding, thus having favourable safety profiles. Here we report the synthesis of a novel compound, trehalose octasulfate and the assessment of its anticoagulation potential. Molecular docking of trehalose and trehalose octasulfate with antithrombin showed a specificity switch in binding affinity on sulfation, where trehalose octasulfate interacts with critical residues of AT that are either directly involved in heparin binding or in the conformational rearrangement of AT on heparin binding. An *in vitro* analysis of trehalose octasulfate demonstrated prolonged clotting time. Lead compound when intravenously injected in occlusion induced thrombotic rats showed remarkable reduction in the size and weight of the clot at a low dose. Delay in coagulation time was observed by analysing blood plasma isolated from rats preinjected with trehalose octasulfate. A decrease in Adenosine 5′-Diphosphate (ADP) induced platelet aggregation indicated a probable dual anticoagulant and antiplatelet mechanism of action. To summarize, this study presents trehalose octasulfate as a novel, effective, dual acting antithrombotic agent.

## 1. Introduction

Thromboembolic disorders that include deep vein thrombosis (DVT) and pulmonary embolism (PE) have an annual incidence of approximately 1 in 1000 in western populations [1]. Thrombosis underlies one of the most frequent causes of cardiovascular diseases like myocardial infarction and stroke [2]. Further, venous thrombosis has been reported to be the second leading factor of death in cancer patients and is a major cause of morbidity and mortality during pregnancy and child birth. Besides the acute morbidity, thrombosis is generally followed by post-thrombotic complications with patients being prone to recurrent episodes of crippling pain and skin ulceration. In addition to the mortality risk, hospitalization is prolonged in patients with DVT and PE as a ramification of any surgical or medical disease, leading to increased healthcare expenditure [3, 4]. The first line in the management of venous thromboembolism (VTE) is to restrict the extension of thrombus so as to reduce the risk of PE and minimize the post-thrombotic complications [5]. This is mainly achieved by the use of anticoagulants, with heparins and coumarins being the most widely used. Even with the remarkable success in managing thrombotic events, the long-term use of heparins and coumarins is associated with a number of drawbacks which chiefly include unavoidable risk of bleeding, narrow therapeutic window, variable patient response and various other complications owing to their non-specific binding, food and drug interactions and immunologic reactions [6–8]. The introduction of new anticoagulants based on low molecular weight heparins (LMWH) and a minimal antithrombin (AT) binding pentasaccharide sequence, for example, fondaparinux, dabigatran, and rivaroxaban, has successfully overcome certain limitations posed by heparin therapy. However, they still are associated with bleeding risk and lack an effective antidote to reverse excessive anticoagulation. Further, their synthesis is a complex, multistep and low-yield procedure [9–12]. Thus, the drawbacks and limitations of current antithrombotic agents have prompted a search for new antithrombotic drugs with reliable safety profiles and feasible synthetic procedures. Towards the design of new oral or intravenous molecules with anticoagulant properties as an alternative to heparin therapy, scaffolds with less anionic and more hydrophobic nature are anticipated to have reduced non-specific interactions compared to heparin. Several efforts in this direction have yielded the synthesis of heparin and heparan sulfate mimetics that mimic the binding of heparin to anticoagulant proteins like AT and heparin cofactor II (HCII) and/or to procoagulant factors, thrombin (fIIa) and factor Xa (fXa) [13]. These molecules include sulfated flavonoids [14–18], benzofurans [19], sulfated dehydrogenation polymers (DHPs) of lignin type [20], isoquinolines [21], and xanthones [22]. In addition, oligosaccharides, namely, dermatan sulfate hexasaccharides, and sulfated bis-lactobionic and bis-maltobionic acid amides have been reported to inhibit fIIa via the activation of its endogenous inhibitor, HCII [23–25]. Lately, another sulfated disaccharide, sucrose octasulfate was shown to exhibit anticoagulant action through a HCII-dependent thrombin inhibition and has been used as an alternative to heparin, yet it is unclear whether it is an effective heparin mimic in its interaction with thrombin [26]. Owing to their structural diversity and hydrophobic nature, better modulators are expected from these scaffolds and compared to heparins, these small sulfated compounds are increasingly gaining importance as inhibitors of coagulation [14–18, 27, 28].

Here, we aimed to synthesize a new small sulfated molecule based on a saccharide skeleton and test its antithrombotic potential. On the basis of molecular docking based screening of small sugars, we selected disaccharide trehalose for sulfation. We report the synthesis and characterization of novel trehalose octasulfate and the assessment of its* in vitro* and* in vivo* anticoagulant properties. The anticoagulant activity of trehalose octasulfate showed a 2-3-fold prolongation of activated partial thromboplastin time (APTT) and prothrombin time (PT) at micromolar range, indicating its promising role in delaying coagulation. The effect of the test compound on thrombus formation was determined in an occlusion induced thrombosis model and by monitoring the clotting times in the blood plasma isolated from rats preinjected with trehalose octasulfate. We observed a remarkable reduction in the thrombus size and weight in the rats preinjected with trehalose octasulfate. Further, the clotting tests of the plasma isolated from the trehalose octasulfate preinjected animals showed a delay in coagulation time. A decrease in ADP induced platelet aggregation in the presence of trehalose octasulfate indicated its dual mechanism of action.

## 2. Materials and Methods

### 2.1. Docking

Autodock Vina (that employs the iterated local search global optimizer for global optimization for local minima search) [29] was used to find the relative affinity of trehalose and trehalose octasulfate with antithrombin. AT (PDB ID:1E05) and HCII (PDB ID:1JMJ) were processed in Autodock tools (ADT) [30], all water molecules were removed, polar hydrogens were added, Kollman charges were assigned to all atoms, and Gasteiger charges were calculated. The ligand PDB files were also processed in ADT. Polar hydrogens were added and Gasteiger charges were calculated and the rigid root and rotable bonds were defined by the Autotors tool of ADT. Blind docking was performed with affinity grid maps of 62 × 58 × 60 points (for IJMJ) and 62 × 62 × 56 points (for IE05) and 1.00 Å grid point spacing centered on whole protein encompassing the heparin binding domain (HBD) using the autogrid tool of ADT. We have considered the minimum energy conformation state of both ligands, showing binding affinity in kcal/mol. Images of ligand and receptor bound complexes were prepared in Ligplot visualizing program [31] and polar contacts between them were noted down.

### 2.2. Chemistry

Triethylamine sulfur trioxide adduct was purchased from Sigma-Aldrich and trehalose from MP-Biomedicals. The solvents used were of HPLC grade procured from Sigma-Aldrich.Precoated aluminium sheets (Silica gel 60 F_254_, Merck Germany) were used for thin-layer chromatography (TLC) and spots were visualized using 5% H_2_SO_4_ in methanol as the developing reagent. Purity of the compound was checked by UHPLC on UHPLC-ELSD-MS Agilent 3100 MS using Acquity UPLC BEH C18 Column (1.7 *μ*m, 2.1 mm × 50 mm I.D). The mobile phases were degassed for 15 min before use. 5 mM ammonium acetate in water and acetonitrile was used as mobile phase at a flow rate of 0.6 mL/min with detection at 214 nm. The sample was prepared in methanol + acetonitrile + water mixture (1 : 1 : 1). The IR spectra of compounds were taken on Agilent Cary 630 FT-IR spectrometer. ^1^H-NMR and ^13^C-NMR spectra were obtained at ambient temperature using a Bruker Spectrospin DPX-400 MHz NMR instrument in D_2_O using tetramethylsilane (TMS) as an internal standard. Splitting patterns are designated as follows: s, singlet; d, doublet; t, triplet; m, multiplet. Chemical shift values are given in parts per million (ppm). Mass spectra were recorded on a Q Star XL hybrid electron spray ionization high resolution mass spectrometer (Applied biosystems) in a scan range of 100 to 1000 atomic mass units (amu). Melting point was recorded on a digital Buchi melting point apparatus (M-560) and was reported uncorrected.

#### 2.2.1. Synthesis of Trehalose Octasulfate

**Scheme 1:**
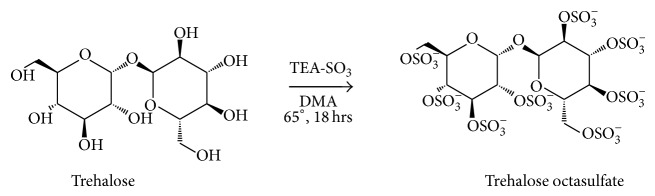


Modification was done using previously reported method [27]. In a solution of trehalose (378 mg, 1 mmol) in DMA (15 mL), triethylamine-sulfur trioxide adduct (4 equiv/OH, 5.8 gm, 32 mmol) was added and the reaction mixture was stirred overnight at 65°C (Scheme 1). After completion of the reaction as checked by TLC, the reaction mixture was poured into 150 mL cold acetone under basic conditions (a few drops of triethylamine were added) and left at 4°C for 24 hours. On the next day, acetone was removed under vacuum and the crude oil was washed with acetone and diethyl ether (3 times). The oil was then dissolved in 30% sodium acetate aqueous solution and the suspension obtained was precipitated in ethanol.

*(1) Trehalose 3, 3*′*, 4, 4*′*, 5, 5*′*, 6, 6*′*-O-Octasulfate*. Creamy white solid; melting point: 210–215°C; yield: 60% IR (neat): 1637, 1229, 951, 808, 894, 1032 cm^−1^; ^1^H-NMR (D_2_O, 400 MHz) (*δ*, ppm): 5.49 (d, 2H), 4.43-4.20 (m, 12 H); ^13^C-NMR (D_2_O, 100 MHz) (*δ*, ppm): 92.18, 75.84, 74.42, 73.69, 68.61, 65.92; ESI-MS (*m*/*z*): 713.5, 487.2, 283.2, 255.2.

### 2.3. Coagulation Assays

Human blood was collected from healthy individuals without any history of bleeding, thrombosis, or consumption of medication known to alter blood coagulation for 2-3 weeks prior to collection. Venous blood collected in citrated vials containing 3.8% sodium citrate solution was centrifuged for 20 min at 2400 g to separate the plasma. The supernatant plasma separated from the cell debris was pooled and used immediately for coagulation time measurements. The clotting assays were performed using commercial kits according to the manufacturer's instructions. Clotting time kits used for APTT (00597) and TT (00611) were from C.K. Prest, France, and PT kit (00667) was from Neoplastine C1 Plus. Trehalose and trehalose octasulfate were dissolved in phosphate sodium EDTA (PNE) buffer. The working concentration of trehalose in the clotting assays ranged from 0 to 10^−3 ^M and 0 to 1^−3^ M for trehalose octasulfate. For APTT assay, 100 *μ*L citrated plasma was mixed with 100 *μ*L of test compound or buffer solution for test and control reactions respectively and incubated at 37°C for 1 minute; 200 *μ*L of APTT reagent was then added and the mixture was again incubated at 37°C for 4 minutes. 200 *μ*L of 20 mMol/L CaCl_2_ was then added and tubes were monitored for clotting time. For PT assay, 100 *μ*L citrated plasma was mixed with 100 *μ*L of buffer or test solution for control and test reactions respectively and incubated for 1 minute at 37°C; 200 *μ*L of PT reagent was then added and the time taken for clot formation was noted. TT test was carried out by mixing 100 *μ*L citrated plasma with 100 *μ*L of buffer or test solution for control and test reactions respectively and incubating at 37°C for 1 minute, followed by the addition of 200 *μ*L of TT reagent. Time taken for clot formation was noted. All the tests were performed in triplicate and repeated at least three times. PNE was taken in control sets to correct the buffer contributions and was compared with the values of coagulation times of plasma alone. Coagulation time prolonging ratio was calculated by comparing the clotting time in the presence of test compound with that when buffer was used in place of test.

### 2.4. *In Vivo* Studies

Sprague Dawley rats (200–250 g) were used for the study. Animals were maintained in polypropylene cages under an ambient temperature of 25°C ± 1°C and a relative humidity of 45% to 55% in a hygienic environment under 12:12 hours light-dark cycle. The animals had free access to food pellets and purified water. All the experimental procedures were approved by the animal ethics committee of Jamia Millia Islamia, New Delhi, India.

Sprague Dawley rats were divided into four groups (Table 1). The test compounds were preinjected to the experimental animals by tail vein injection method [32, 33]. Following the injection, all the animals in the G-A to G-D underwent inferior vena caval (IVC) ligation for the thrombus to be inducted by flow restriction approach. This approach provides a total stasis environment and results in a very severe vein wall reaction to thrombosis [34]. Animals were anesthetized and IVC exposed through a midline laparotomy by dissecting at the level of renal veins. The infrarenal IVC was identified and ligated below the renal veins until the iliac trunk (1–1.2 cm in length) with nonreactive 7-0 prolene suture. After 24 hours, animals were euthanized and thrombosed inferior vena cava was harvested. Since thrombus size is a direct measure of clot formation and dissolution, the following parameters were recorded in all the animals: (i) total length of the ligated IVC, (ii) length and (iii) weight of the thrombus formed inside the ligated IVC. Using these directly measurable quantitative parameters, the ratio of the weight of the thrombus (mg) to the length of the IVC ligation (mm) were calculated as

(1) Ratio (Weight  to  Length) =Weight of Thrombus formed mgLength of IVC ligation mm.

The quantitative parameters obtained in test compound injected groups (G-C and G-D) (Table 1) were compared with corresponding positive controls (G-A and G-B) (Table 1). In the initial phase of experiments, dosage regimen was determined by injecting the compounds at varying dosages of 10 mg/kg, 5.0 mg/kg, 2.5 mg/kg and 0.5 mg/kg body weight of rat. Among these doses, 2.5 mg/kg of body weight of trehalose octasulfate yielded quantifiable amounts of vein wall tissue and thrombus and was thus set as an optimum dosage to study its effect on thrombus formation. At the time of euthanasia blood was collected from retro-orbital plexus of animals. Plasma was isolated and APTT and PT assays were performed.

### 2.5. Platelet Aggregation Assay

The antiplatelet activity of test compounds was determined by whole blood aggregation. Platelet aggregation was monitored in whole blood by using a Lumi-Aggregometer (Chrono-log model 700, USA) as per manufacturer's instructions. Platelet aggregation was measured in freshly collected whole blood with constant stirring at 1200 rpm. ADP was used as the agonist. The maximum impedance (ohms) was recorded as a measure of platelet aggregation in whole blood. The experiment was repeated with blood collected from the compound treated rats (Table 1) and the impedance of aggregation of these experimental animal groups was compared to that of vehicle control ones to study the effect of trehalose octasulfate on agonist induced platelet aggregation response.

## 3. Results and Discussion

### 3.1. Docking Studies

**Figure 1:**
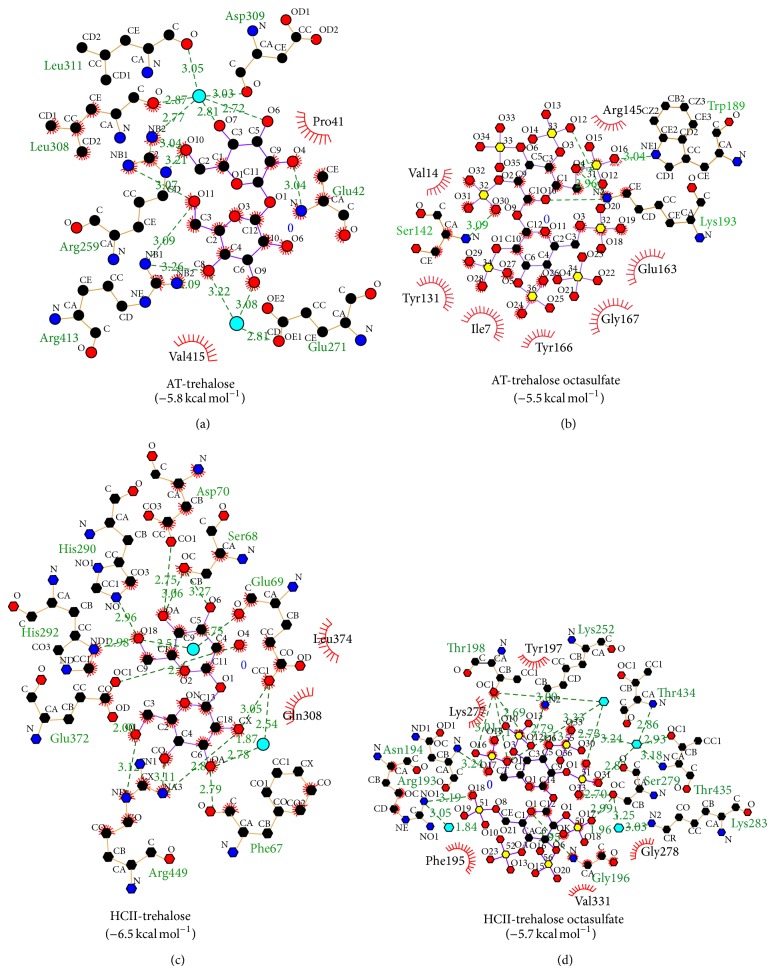
Ligplot analysis of binding of trehalose and trehalose octasulfate to antithrombin (1E05) heparin cofactor II (1JMJ).

Molecular docking study of trehalose and trehalose octasulfate was carried out to check their relative affinity towards the target proteins AT and HCII. AT and HCII are the most critical endogenous anticoagulant molecules that regulate coagulation by inhibiting procoagulant proteases, namely, thrombin, fXa, fIXa, and fXIa (AT) and thrombin (HCII). Both AT and HCII require heparin as a cofactor to inhibit these proteases at physiologically relevant rates, underlying the principal use of heparin as anticoagulant [35–37]. Heparin binding domain of AT and HCII comprises of positively charged residues of the helix D of both AT and HCII and helix A of AT [38]. In consistency with our earlier study [39], we observed a switch in the specificity of binding affinity of trehalose on sulfation. We observed that trehalose octasulfate, apart from interacting with AT residues, Glu163, Tyr166, Trp189, and Lys193, interacts with some critical residues (Ile7, Tyr131, Val141, Ser142, Arg145, and Gly167) (Figure 1) that are involved either in heparin binding or in the conformational rearrangement of AT on heparin binding. There are several reports that support the involvement of AT residues 41–49 and 124–145 in heparin binding [40], other residues have been implicated as well, for example, natural variant Ile7Asn is associated with decreased heparin binding indicating its involvement in heparin based modulation of AT [41]. Arg145 is placed within the heparin binding site of human AT [42]. Further, in native AT, Tyr131-Asn127-Leu130-Leu140-Ser142 forms a tight cluster at the helix D-strand 2A interface and tight interactions between Tyr131 and neighbouring hD, and s2A stabilizes the native conformation of AT; it has also been hypothesized and tested that disrupting this cluster would activate AT independently of heparin [43]. Helix D residues 120–124 make multiple van der Waals contacts with residues 161–166 of helix E, where the rotation of helix D on binding to heparin pivots on the side chain of Phe123 and clashes into helix E. Here, the movement of Tyr166 is considered one of the important events in the propagation of conformational change from the heparin binding site to the distant hinge region of the RCL, required for the allosteric activation of AT [44]. Thus on the basis of the interaction profile of trehalose octasulfate with AT residues, Ile7, Tyr131, Val141, Ser142, Arg145 and Gly167, we speculated that it may modulate AT-based coagulation and hence set forth for its synthesis.

### 3.2. Synthesis of Trehalose Octasulfate

Trehalose octasulfate was synthesised from its precursor in moderate yield in one step reaction as shown in Scheme 1. Purity of the compound was checked by UHPLC and the structure was confirmed by FT-IR, ^1^H-NMR, ^13^C-NMR, and mass analyses. In IR analysis, absence of characteristic bands at 3400–3150 cm^−1^ corresponding to OH group indicates that all the hydroxyl groups have undergone sulfation. In addition to this, two strong bands at 1229 cm^−1^ corresponding to S=O stretch indicated the presence of sulfate group. The modification was further confirmed by ^1^H-NMR spectral analysis, in which all the protons of sugar ring were found deshielded in the range 5.49–4.20 ppm in comparison to unmodified trehalose (5.17–3.40). All the OH protons of trehalose disappeared in modified trehalose establishing the formation of octasulfated trehalose. In ^13^C-NMR spectra, carbons to which sulfate group is attached in the modified product were found to be deshielded as compared to trehalose providing confirmatory evidence for the substitution of –OH group by sulfate group. It has been reported that electron ionization (EI) of certain scaffolds involves complex fragmentation due to the broad spectra of internal energy of the molecular ion peak (M^+∙^) which suppresses the M^+∙^ and other primary fragments containing the structural information [45]. In our case as well, no distinct molecular ion peak was obtained in modified trehalose that may be due to fragmentation of sulfated trehalose into various fragment ions.

### 3.3. *In Vitro* Clotting Assays

**Figure 2:**
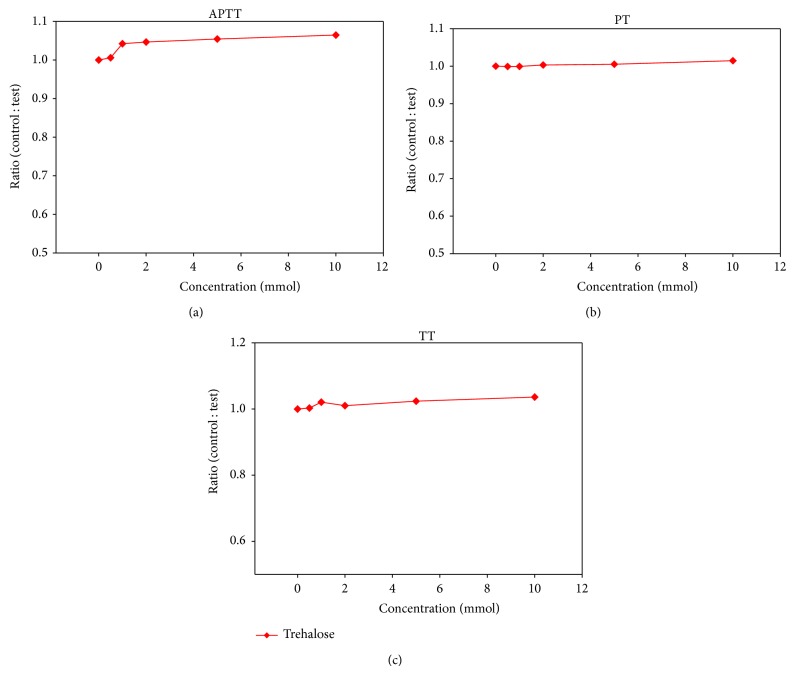
Effect of trehalose on clotting assays (a) APTT, (b) PT and (c) TT using human pooled plasma, expressed as ratio of clotting time in the presence and absence of trehalose octasulfate. Values represent an average of three independent experiments.

**Figure 3:**
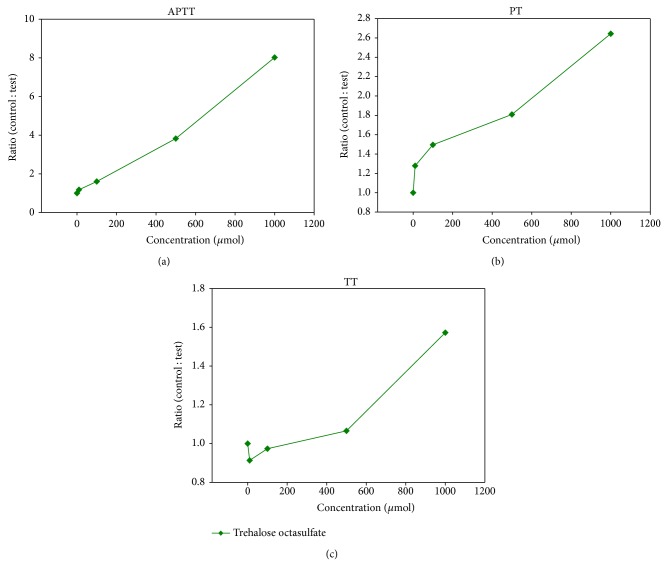
Effect of trehalose octasulfate on clotting assays (a) APTT, (b) PT and (c) TT using human pooled plasma, expressed as ratio of clotting time in the presence and absence of trehalose. Values represent an average of three independent experiments.

To assess the anticoagulant property of trehalose octasulfate, the* in vitro* anticoagulant activity was analyzed for both sulfated and nonsulfated trehalose in human plasma by the three conventional coagulation assays, APTT, PT, and TT and the results are summarized in Figures 2 and 3. These results were expressed as ratio of clotting time in the presence or absence of test compound to find out the extent of modulation with respect to the control. We observed that the ratio of the coagulation times in the presence of the nonsulfated compound with that of control plasma was close to 1 in all the three clotting tests, indicating that trehalose does not alter the coagulation pathways (at 10 mM) (Figure 2). However, the sulfated derivative exhibited promising anticoagulant properties in a dose-dependent manner. Trehalose octasulfate prolonged the clotting times APTT and PT significantly (Figure 3). At 1 mM concentration of trehalose octasulfate, there was an approximately 8-fold increase in APTT and 3-fold increase in PT. Preliminary information about the mode of action of these lead compounds could be inferred based on the varying effect on clotting assays, since each assay gives interaction of different stages of the coagulation pathway [46]. In view of the fact that trehalose octasulfate prolonged APTT and PT (APTT more than PT), its probable mode of action may be linked to the common pathway and can also be implicated in the intrinsic pathway. Further, in order to set an optimum anticoagulating dosage of the lead compound, we analysed the concentration required to double the coagulation times (APTT_2_ and PT_2_) (Table 2). The rationale was to find an effective concentration that only prolongs/delays clotting to a limited and controlled extent so as not to shift the equilibrium towards bleeding.

### 3.4. *In Vivo* Antithrombotic Effect

**Figure 4:**
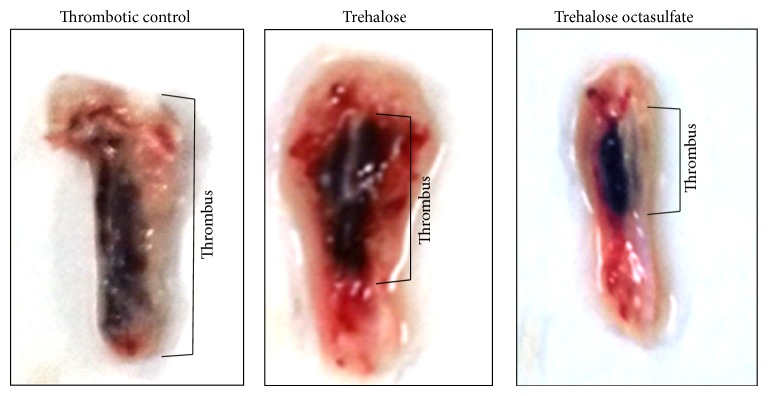
Comparison of thrombi harvested from control, trehalose and trehalose octasulfate treated thrombosis model animals.

**Figure 5:**
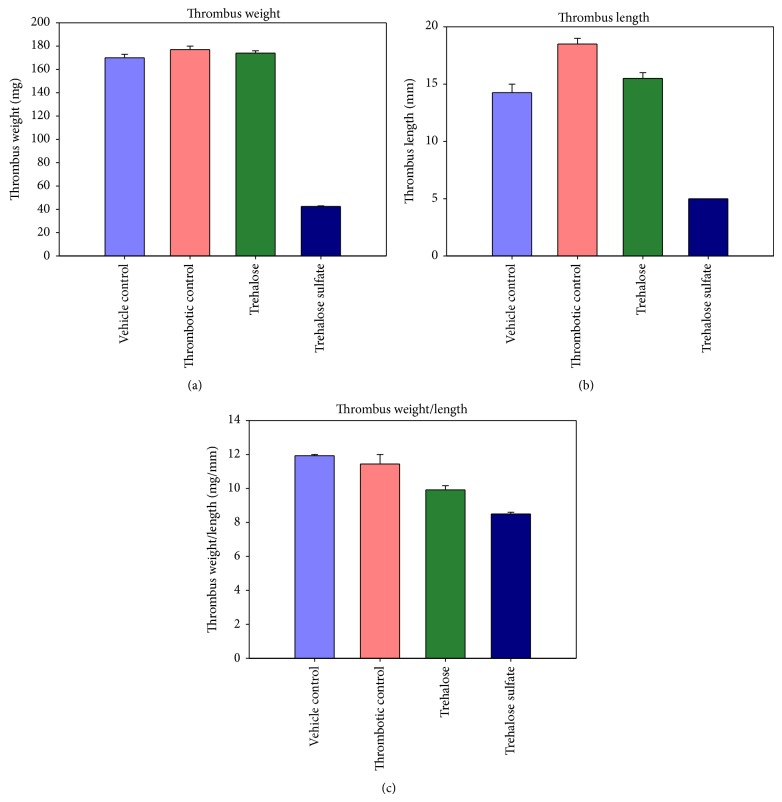
Comparison of thrombi from control, trehalose and trehalose octasulfate treated thrombosis model animals; (a) weight, (b) length, and (c) weight/length ratio of the thrombus formed inside the ligated IVC.

In our study, inferior vena caval ligation caused prominent thrombus formation in the occluded region of the vena cava in Sprague Dawley rats. Control (thrombotic) animals developed a prominent thrombus exhibited with an increased length and weight of the thrombus and were associated with a significant increase in the derived parameter, ratio of thrombus weight to IVC length. The thrombus harvested from the vehicle control group of rats (vehicle or buffer injected) was also considerably prominent, with dimensions close to the thrombotic control (no treatment). The preinjection with trehalose octasulfate (2.5 mg/kg body weight) caused a marked decrease in thrombus size, as evident by the prominent reductions in the thrombus dimensions as compared to the corresponding control group (Figures 4 and 5). Figures 4 and 5 show the antithrombotic effect of trehalose octasulfate, an appreciable reduction in thrombus weight and length in the trehalose octasulfate treated rat as compared to the thrombotic control rat was observed. A consequent decrease in thrombus weight/length ratio as compared to the thrombotic rat could be observed (Figure 5(c)). These results clearly indicate the* in vivo* antithrombotic potential of trehalose octasulfate. Thus, in addition to* ex vivo *anticlotting potency, trehalose octasulfate was found to possess* in vivo* antithrombotic activity, as evident by the significant inhibition of occlusion-induced venous thrombosis in rats injected with this compound. Further, while establishing the optimum dosage regimen, trehalose octasulfate was able to completely inhibit thrombus formation at the highest dose tested (10 mg/Kg body weight). This further ascertained a dose-dependent effect of trehalose octasulfate, which may enable to set its optimum dosage.

### 3.5. *In Vivo* Clotting Assays

**Figure 6:**
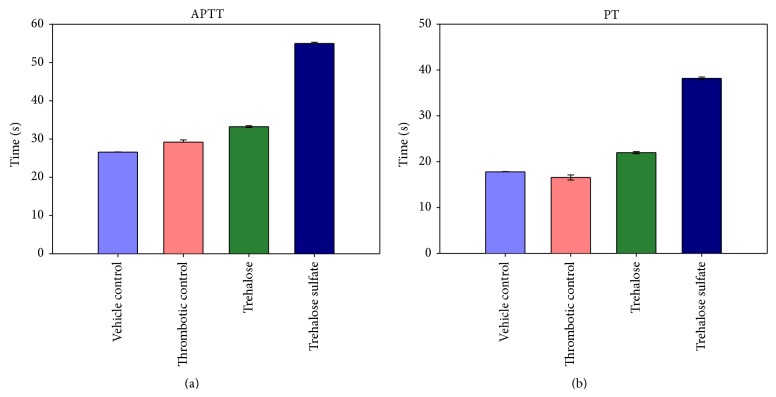
Effect of trehalose and trehalose octasulfate on (a) APTT and (b) PT in rat plasma after IV infusion of the compounds.

In consistency with the* in vitro* anticoagulating effect of trehalose octasulfate, coagulation parameters APTT and PT were prolonged* in vivo* as well. Plasma isolated from the trehalose octasulfate injected rats showed a delayed coagulation in both the intrinsic and extrinsic pathways at the optimum antithrombotic dosage.  Figure 6 shows the APTT and PT profile of trehalose octasulfate treated rat. Approximately 2-fold rise in both the clotting times was observed as compared to both the control plasma and the plasma isolated from ligated (thrombotic) animal.

### 3.6. Antiplatelet Effect

**Figure 7:**
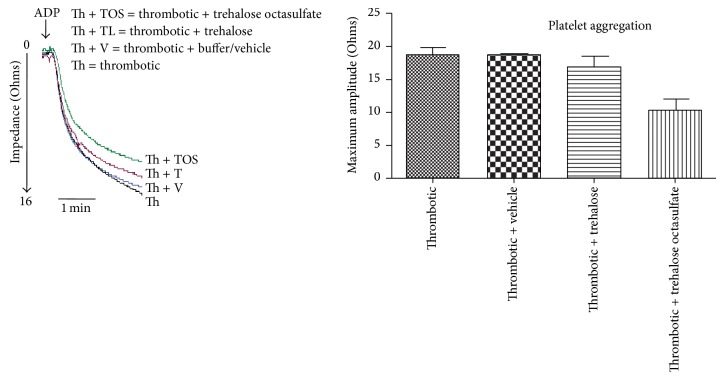
Effect of trehalose octasulfate on platelet aggregation, in whole blood of rat injected with the compounds, *P* < 0.05.

Whole blood aggregation study was conducted to evaluate the efficacy of trehalose octasulfate on platelet aggregation. Trehalose octasulfate appreciably decreased the impedance of ADP induced platelet aggregation.  Figure 7 shows a representative platelet aggregation curve of trehalose octasulfate treated animals, measured in freshly collected whole blood. A decrease in platelet aggregation in blood collected from trehalose octasulfate injected animals was observed. Therefore, platelet aggregation studies revealed the antiplatelet activity of trehalose octasulfate, suggesting a dual mechanism of action in modulating coagulation.

## 4. Conclusion

Trehalose octasulfate in the present study showed a significant and dose-dependent retardation of intrinsic and extrinsic coagulation pathways both* in vivo* and* ex vivo*. Unmodified/nonsulfated trehalose did not affect coagulation, indicative of sulfation being an ideal strategy to impart anticoagulant properties in natural scaffolds. Trehalose octasulfate also showed promising antithrombotic property against IVC statis-induced venous thrombosis model in rats, which is one of the most efficient models for screening antithrombotic activity of the drugs. Further, the antiplatelet activity of trehalose octasulfate in terms of ADP induced platelet aggregation indicated their antiplatelet activity. Thus, from the mechanistic point, trehalose octasulfate employs a multitarget strategy to modulate the activity of multiple components of thrombus formation, suggesting a dual mechanism of action. However, the molecular details of its role in reducing the coagulation need to be completely understood.

## Figures and Tables

**Table 1:** Experimental groups considered for *in vivo* antithrombotic studies.

Experimental group	Treatment
A	Vehicle control
B	Thrombotic control (occluded)
C	Trehalose injected
D	Trehalose octasulfate injected

**Table 2:** Concentration of trehalose octasulfate required to double the clotting times.

Compound	APTT_2_	PT_2_
Trehalose octasulfate	400 *μ*M	900 *μ*M

## Abbreviations

AT: Antithrombin
HCII: Heparin cofactor II
ADP: Adenosine 5′-Diphosphate
HBD: Heparin binding domain
DVT: Deep vein thrombosis
PE: Pulmonary embolism
VTE: Venous thromboembolism
LMWH: Low molecular weight heparin
fIIa: Thrombin
fXa: Factor Xa
DHPs: Dehydrogenation polymers
APTT: Activated partial thromboplastin time
PT: Prothrombin time
TLC: Thin-layer chromatography
DMA: Dimethylacetamide.
